# Supplementary material for: Comparing labour induction outcomes using misoprostol and dinoprostone in term pregnancies: A retrospective study at Kiambu Level 5 Hospital between 2018 and 2020
Source: PLoS One. 2024 May 31;19(5):e0304631. doi: 10.1371/journal.pone.0304631 (PMC11142478; doi:10.1371/journal.pone.0304631)
Supplement: S2 File — (PDF) [file pone.0304631.s002.pdf]

Qualitative results regarding the impact of the newly formed MPDSR committee

Theme 1: Team Work

Participants felt that one of the main benefits of the institutionalisation of MPDSR was improved teamwork through better information flow. MPDSR presented a forum for better feedback, guideline creation and distribution, service delivery audits and gap identification with resultant rectification.

30% of respondents referred to the teamwork developed by MPDSR as beneficial. 50% did not find that there were any challenges associated with teamwork. This figure rose to 88% for those with less than 4 years' experience working in the department.

Of note is that those who had less than 1 year experience working did not see any effect of the MPDSR in general, did not think it helped enhance communication between cadres nor between seniors and juniors, and felt it did not add any benefit to the documentation. This group was also most likely to use Misoprostol for clients who could not afford dinoprostone.

Theme 2: Method of Correction

Respondents felt that the methods used for correction were "instilling fear" and "intimidation" which would lead to "defensive responses". To the question "In your opinion, what have been the main challenges brought about by the MPDSR committee in Kiambu level 5 hospital?" respondents stated:

"*Sometimes nurses feel intimidated where the MPDSR committee concentrates on individuals who attended to the patients instead of identified gaps*" **(Respondent_2)**

"*Bringing some changes that are not sustainable*" **(Respondent_11)**

“*People refusing to accept change and change with change*” **(Respondent_1)**

100% of all respondents reported that before the MPDSR was started the situation was worse than it is now. When asked if MPDSR had influenced communication between cadres, 41 % of respondents reported a positive effect with another 41% documenting no effect. 16% felt that communication between cadres had gotten worse. With regards to communication between seniors and juniors, 50% of respondents reported that MPDSR had had some positive effect, 33% said it had no effects with 16% noting that things were worse.
